# Supplementary material for: HIVprotI: an integrated web based platform for prediction and design of HIV proteins inhibitors
Source: J Cheminform. 2018 Mar 9;10:12. doi: 10.1186/s13321-018-0266-y (PMC5845081; doi:10.1186/s13321-018-0266-y)
Supplement: Supplementary file 2 — Additional file 2. Source code of HIVProtI web server. [file 13321_2018_266_MOESM2_ESM.zip › HIVprotI_Source-code/hivprotI/weka-3-6-6/doc/weka/gui/ExtensionFileFilter.html]

ExtensionFileFilter


---


|  |  |  |
| --- | --- | --- |
| **Overview** \| **Package** \| **Class** \| **Tree** \| **Deprecated** \| **Index** \| **Help** \| **Weka's home** | |  |
| **PREV CLASS**   **NEXT CLASS** | **FRAMES**    **NO FRAMES**     **All Classes** |  |
| SUMMARY: NESTED \| FIELD \| CONSTR \| METHOD | DETAIL: FIELD \| CONSTR \| METHOD |  |


---


## weka.gui Class ExtensionFileFilter

```
java.lang.Object
  javax.swing.filechooser.FileFilter
      weka.gui.ExtensionFileFilter
```

**All Implemented Interfaces:**: java.io.FilenameFilter, java.io.Serializable

---

``` public class ExtensionFileFilter extends javax.swing.filechooser.FileFilter implements java.io.FilenameFilter, java.io.Serializable ```

Provides a file filter for FileChoosers that accepts or rejects files
based on their extension. Compatible with both java.io.FilenameFilter and
javax.swing.filechooser.FileFilter (why there are two I have no idea).

**Version:**
:   $Revision: 1.8 $

**Author:**
:   Len Trigg (trigg@cs.waikato.ac.nz)

**See Also:**: Serialized Form

---

**Constructor Summary**

`ExtensionFileFilter(java.lang.String[] extensions,
java.lang.String description)`
  
          Creates an ExtensionFileFilter that accepts files that have any of
the extensions contained in the supplied array.

`ExtensionFileFilter(java.lang.String extension,
java.lang.String description)`
  
          Creates the ExtensionFileFilter


| **Method Summary** | |
| --- | --- |
| `boolean` | `accept(java.io.File file)`             Returns true if the supplied file should be accepted (i.e.: if it has the required extension or is a directory). |
| `boolean` | `accept(java.io.File dir, java.lang.String name)`             Returns true if the file in the given directory with the given name should be accepted. |
| `java.lang.String` | `getDescription()`             Gets the description of accepted files. |
| `java.lang.String[]` | `getExtensions()`             Returns a copy of the acceptable extensions. |

**Methods inherited from class java.lang.Object**

`equals, getClass, hashCode, notify, notifyAll, toString, wait, wait, wait`

**Constructor Detail**

### ExtensionFileFilter

```
public ExtensionFileFilter(java.lang.String extension,
                           java.lang.String description)
```

:   Creates the ExtensionFileFilter

    **Parameters:**: `extension` - the extension of accepted files.: `description` - a text description of accepted files.

---


### ExtensionFileFilter

```
public ExtensionFileFilter(java.lang.String[] extensions,
                           java.lang.String description)
```

:   Creates an ExtensionFileFilter that accepts files that have any of
    the extensions contained in the supplied array.

    **Parameters:**: `extensions` - an array of acceptable file extensions (as Strings).: `description` - a text description of accepted files.


**Method Detail**

### getDescription

```
public java.lang.String getDescription()
```

:   Gets the description of accepted files.

    :   **Specified by:**: `getDescription` in class `javax.swing.filechooser.FileFilter`
    :   **Returns:**: the description.

---


### getExtensions

```
public java.lang.String[] getExtensions()
```

:   Returns a copy of the acceptable extensions.

    :   **Returns:**: the accepted extensions

---


### accept

```
public boolean accept(java.io.File file)
```

:   Returns true if the supplied file should be accepted (i.e.: if it
    has the required extension or is a directory).

    :   **Specified by:**: `accept` in class `javax.swing.filechooser.FileFilter`
    :   **Parameters:**: `file` - the file of interest. **Returns:**: true if the file is accepted by the filter.

---


### accept

```
public boolean accept(java.io.File dir,
                      java.lang.String name)
```

:   Returns true if the file in the given directory with the given name
    should be accepted.

    :   **Specified by:**: `accept` in interface `java.io.FilenameFilter`
    :   **Parameters:**: `dir` - the directory where the file resides.: `name` - the name of the file. **Returns:**: true if the file is accepted.


---


|  |  |  |
| --- | --- | --- |
| **Overview** \| **Package** \| **Class** \| **Tree** \| **Deprecated** \| **Index** \| **Help** \| **Weka's home** | |  |
| **PREV CLASS**   **NEXT CLASS** | **FRAMES**    **NO FRAMES**     **All Classes** |  |
| SUMMARY: NESTED \| FIELD \| CONSTR \| METHOD | DETAIL: FIELD \| CONSTR \| METHOD |  |


---
